# Anaerobic Codigestion of Municipal Wastewater Treatment Plant Sludge with Food Waste: A Case Study

**DOI:** 10.1155/2016/8462928

**Published:** 2016-09-05

**Authors:** Zubayeda Zahan, Maazuza Z. Othman, William Rajendram

**Affiliations:** ^1^School of Civil, Environmental and Chemical Engineering, Royal Melbourne Institute of Technology, Melbourne, VIC 3000, Australia; ^2^Western Water, Melbourne, VIC, Australia

## Abstract

The aim of this study was to assess the effects of the codigestion of food manufacturing and processing wastes (FW) with sewage sludge (SS), that is, municipal wastewater treatment plant primary sludge and waste activated sludge. Bench scale mesophilic anaerobic reactors were fed intermittently with varying ratio of SS and FW and operated at a hydraulic retention time of 20 days and organic loading of 2.0 kg TS/m^3^·d. The specific biogas production (SBP) increased by 25% to 50% with the addition of 1%–5% FW to SS which is significantly higher than the SBP from SS of 284 ± 9.7 mL_N_/g VS added. Although the TS, VS, and tCOD removal slightly increased, the biogas yield and methane content improved significantly and no inhibitory effects were observed as indicated by the stable pH throughout the experiment. Metal screening of the digestate suggested the biosolids meet the guidelines for use as a soil conditioner. Batch biochemical methane potential tests at different ratios of SS : FW were used to determine the optimum ratio using surface model analysis. The results showed that up to 47-48% FW can be codigested with SS. Overall these results confirm that codigestion has great potential in improving the methane yield of SS.

## 1. Introduction

Sludge production from municipal wastewater treatment plants (MWTPs) is expected to continue to increase with the increasing number of treatment plants being constructed or upgraded due to the growing population connected to the sewage networks of Australia. The disposal of sludge generated at the MWTPs is a problem of increasing importance, representing up to 50% of the current operating costs of a wastewater treatment plant [1]. In Australia, MWTPs produce approximately 360,000 dry tonnes of stabilised sewage sludge to dispose of which costs about $100 M per year [2, 3]. Hence, water authorities operating these plants in Australia have been actively investigating alternative sustainable and economic sludge management pathways [4]. Although different disposal routes are possible, anaerobic digestion (AD) appears to be the most promising sludge management alternative due to its ability to generate bioenergy by the reduction of the sludge volumes to be disposed of [5–8].

Sewage sludge (SS) contains a high percentage of organic matter (60–70% of the dry matter) and nutrients, and typically comprises primary sludge (PS) and waste activated sludge (WAS) [9, 10]. However, since WAS has low biodegradability; the AD of WAS has low efficiency from both processing and economic standpoints [11]. One of the different strategies to enhance the performance of AD is the codigestion of sludge with other organic wastes as it increases biodegradable organic matter and provides a feedstock with an optimum C/N ratio [1, 4–12]. Among the factors that limit the codigestion are the selection and type of new organic wastes and the transportation cost of cosubstrates to the MWTPs [9–13].

Food wastes (FW) from different sources, for example, residential and commercial, are being produced at an increasing rate due to the growing population and rising living standards [8]. FW is available all year round and accounts for a significant proportion of municipal solid waste. In Victoria Australia, FW contributes 35.6% of the total municipal solid wastes when source separated, usually referred to as organic fraction of munipal solid waste [14]. However, due to its high biodegradability and volatile acids, AD as a single substrate may encounter various potential inhibitors including accumulation of volatile fatty acids' (VFAs) accumulations [15]. Therefore, these FW could be beneficial in anaerobic codigestion for high energy recovery as well as solid waste reduction.

The application of anaerobic codigestion for the treatment of SS has been receiving growing attention for improving the biogas yield, solid destruction, and the production of digestate of a suitable quality to use as a fertilizer [16]. Full-scale applications of anaerobic codigestion of SS with FW can become an environmentally sound renewable energy source by creating opportunities to recover the energy potential from these very low or zero cost FW and obtain the benefit of high organic matter to increase the methane yield. This will result in significantly less biosolids' disposal and a reduction in municipal solid wastes as well as the operating costs of the plants.

Many authors have reported increased biogas yields from the codigestion of SS with different types of food and/or food processing wastes. For example, codigestion of sludge mix with fat, oil and grease (FOG) from a meat processing plant (46% VS added to the feed) increased the methane yield by 60% [17]. Similarly, methane yield was 2.6 times higher when SS was codigested with oil and grease (48% of total VS load) from restaurants [18]. Under mesophilic conditions, the highest methane production rate was observed when FW was mixed in the range of 30–40% VS with SS [16, 19].

An MWTP in Melbourne, Australia, produces about 3600 kg solids/day of which 627 kg is WAS and the remaid is PS. Since, this plant is in the progress of upgrading the existing old anaerobic digestion reactor, the management was interested in assessing the feasibility of the codigestion of sludge with two streams of wastes, namely, grease trap waste collected from food businesses in the area, referred to in this study as FOG, and waste from a food products manufacturing factory at a small ratio. The MWTP interest is to maximise methane yield, enhance solids removal, and maintain or improve biosolids quality.

The aim of this study, therefore, was to assess the effect of the sludge: waste ratio on the biogas yield and the quality of digestate and supernatant nutrient produced under semicontinuous conditions and, to monitor process performance and stability during codigestion experiments.

## 2. Materials and Methods

### 2.1. Characteristics of Substrates and Inoculum

The sludge feedstocks used in this study were thickened PS and WAS collected from Melton Recycled Water Treatment Plant, Victoria, Australia. The PS and WAS were mixed at a ratio consistent with their flow rates such that the final mixed SS's total solids (TS) is 4%. The raw PS and WAS were collected several times while running the experiment and each time they were characterised and mixed as described. The characteristics of different SS samples are reported in Table 1. The SS was stored in a sealed plastic container at 4°C.

The FW used were (i) thickened grease trap, referred to in this paper as GT, obtained from a commercial business that collects FOG from restaurants and food businesses in the western area of Melbourne and (ii) wastes from processed food products manufacturing factory denoted as PF. These FW are mostly comprised cooking oil, butter, cheese, meat, bread, meat fat and bones, mayonnaise, salad dressing, and so forth. The food wastes were collected regularly, homogenised using a high speed homogeniser, and then characterised by the parameters shown in Table 1. The TS of the substrates (SS, PF, and GT) was adjusted such that the AD reactors received a feedstock of consistent TS and chemical oxygen demand (COD) concentration throughout the duration of the experiments. The inoculum used in this experiment was collected from the mesophilic anaerobic digester at Melton wastewater treatment plant. The characteristics of the feedstocks (SS, PF, and GT) and the inoculum are shown in Table 1.

### 2.2. Batch Experiments

Batch tests were performed to determine the biochemical methane potential (BMP) of the individual substrates (SS, PF, and GT) and mixtures of the SS and FW (a mixture of PF and GT at 50 : 50 w/w) at different ratios. The experimental design is shown in Table 2. All the BMP tests were performed in 500 mL glass bottles at 37°C according to the guideline of Angelidaki et al. [20]. Each reactor contained 4000 mg VS with VS_substrate_ : VS_inoculum_ ratio of 0.25. In addition, two reactors received only inoculum as a control. The headspace of the bottles was flushed with nitrogen gas for 2 minutes and the bottles were closed with a rubber Suba-Seal. All batch tests were performed in duplicate. The bottles were kept at 37 ± 1°C in an incubated shaker at a constant rotational speed of 100 rpm. The volume of biogas produced was measured using a water displacement unit and the biogas composition was monitored using gas chromatography. The volume of biogas (or methane) from the control was subtracted from the volume of biogas (or methane) produced in each reactor to obtain the net production of biogas or BMP from the substrates fed into the reactor.

### 2.3. Semicontinuous Experiments

SS was mixed with the wastes from the processed food products manufacturing and/or FOG, at the designated ratio of sludge to waste (SS : PF, SS : GT). The experiments were performed in 500 mL glass reactors, designed to allow feeding and nitrogen flushing simultaneously, at 37 ± 1°C in an incubated shaker at a constant rotational speed of 100. The reactors received the substrates at a concentration of 4% TS and operated at an organic loading rate of 2.0 kg TS/m^3^·d. The experiment comprised duplicate reactors for each condition. The reactors were operated at a sludge retention time of 20 days (equivalent to hydraulic retention time, HRT, in this case) and were fed and wasted once a day. The biogas was collected before feeding the reactors every day. The biogas measurement, feeding, and wasting were done within a 15 min window out of the incubator. The reactors were monitored weekly for biogas quality, and the wastage was analysed every ten days for pH, TS, and VS, total COD (tCOD), and soluble COD (sCOD). The feedstock to the reactors was prepared from different substrates at the ratios shown in Table 2.

### 2.4. Analytical Methods

TS and VS were measured by gravimetric analysis according to the Standard Methods 2540B and 2540E, respectively [21]. tCOD and sCOD were measured according to HACH method 8000. The total phosphorus (TP), total nitrogen (TN), ammonium, and volatile acids (VAs) were measured by colorimetric techniques using a HACH spectrophotometer (Model DR/4000 U) according to the methods 10127, 10072, 10031, and 8196, respectively. The samples were centrifuged (Eppendorf 5702, Germany) at 4.4 rpm for 15 mins and then filtered through 0.45 *μ*m filter paper (mixed cellulose esters membrane filter, Advantec, Japan) to measure the soluble constituents. The measurement of pH was carried out using a calibrated pH meter (ThermoOrion, Model 550A) and alkalinity was measured by the APHA method 2320B.

The volume of biogas was normalised to standard conditions compromising dry gas, standard temperature, and pressure (0°C and 1 bar) according to the method described by Strömberg et al. [22] and the results are presented as norm-litre (L_N_). The headspace was corrected for methane (CH_4_) and carbon dioxide (CO_2_) to 100% according to VDI 4630 (2006) [23]. The composition of the biogas was analysed according to APHA method 2720C using gas chromatography (Varian 450-GC, Varian Australia Pty Ltd., Netherlands) equipped with a packed column (GS-CarbonPLOT 113-3132, 1.5 microns, 30 m*∗*0.320 mm, stainless steel, Agilent Technologies Inc., Australia) and a thermal conductivity detector. The carrier gas used was helium at a flow rate of 28 mL/min. The temperatures of the column, detector, and injector were 70°C, 200°C, and 100°C, respectively. The biogas was collected and manually injected using a 50 mL FORTUNA® Optima glass syringe (Poulten & Graf, Germany). Calibration was done using three points and five levels of CH_4_, CO_2_, and nitrogen (BOC, Australia). Screening of the metals in the digestate samples was tested for sodium (Na) to cerium (Ce) by a commercial laboratory (ALS Environmental Division: Water Research Group).

### 2.5. Statistical Analysis

Predictions of the optimum mixture ration for two and three substrates from batch tests were obtained using MATLAB R2013b. Furthermore, a predictive model for optimum FW incorporation was prepared with surface and contour plots. To determine the significance of difference in cumulative methane yields over the digestion period, each set of codigestion feedstock was statistically analysed with 100% SS using one-way analysis of variance (ANOVA) at *α* = 0.05 in MATLAB R2013b.

## 3. Results and Discussion

### 3.1. Batch Experiments

**Figure 1:**
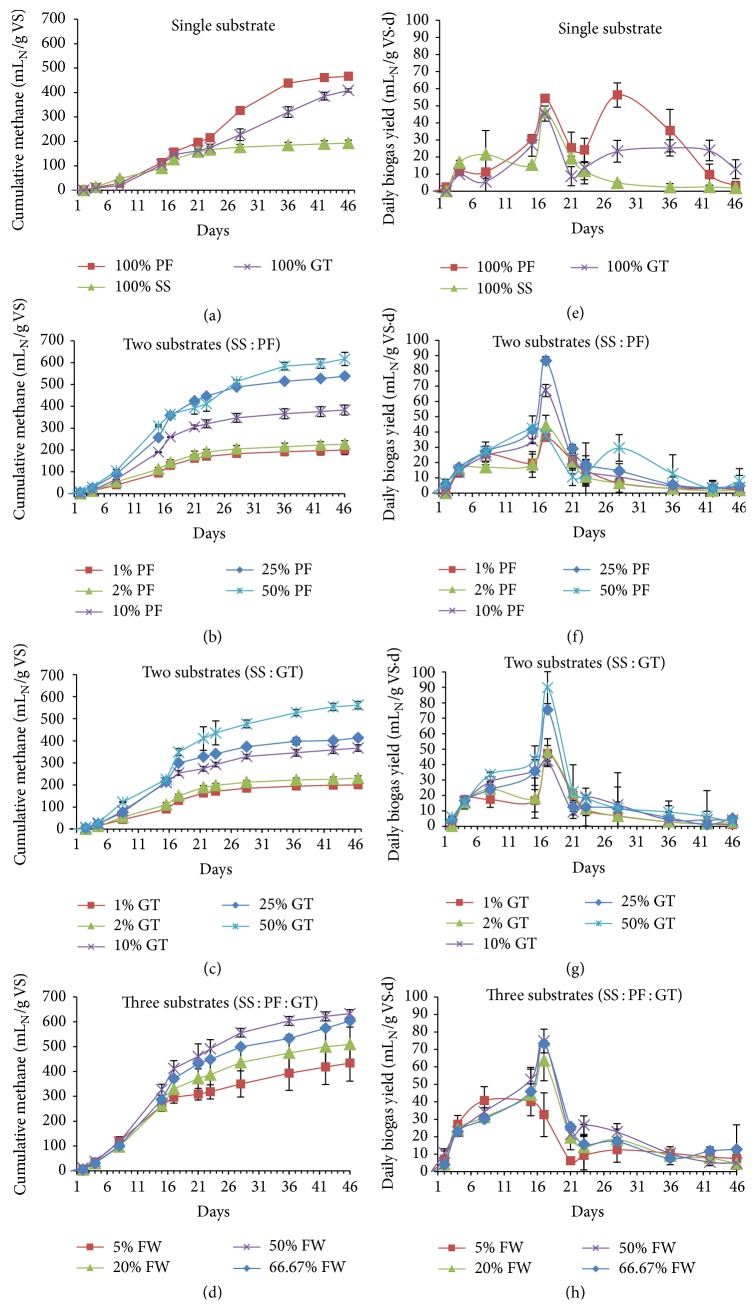
Accumulative methane production (a–d) and daily biogas yield (e–f) from batch experiments of single, two, and three substrates.

Batch experiments were carried out to investigate the optimum ratio of FW for incorporation in SS. The effects of two substrates and three substrates were also investigated at different mixture ratios. The cumulative methane yields and the daily biogas yields during the anaerobic codigestion are shown in Figures 1(a)–1(d) and 1(e)–1(h), respectively. The BMP tests continued for 46 days until little or no biogas production was observed. The results presented are the net biogas and methane yield from the feedstock after subtracting the control yield.

**Figure 2:**
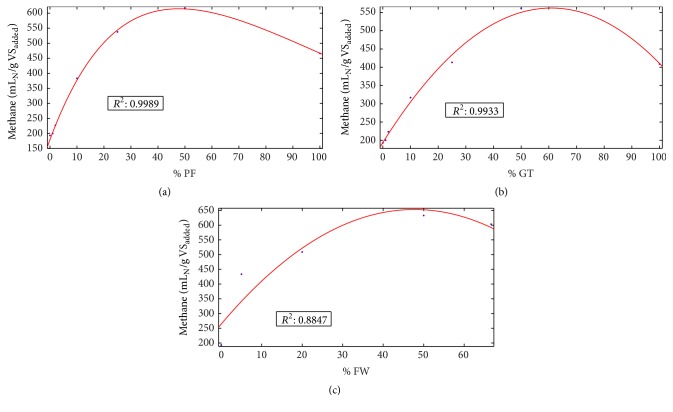
Prediction of optimum SS and FW mix ratio according to the methane yield: (a) %PF, (b) %GT, and (c) %FW.

According to Figure 1(a), the BMP of 100% SS was 192 ± 12.3 mL_N_ CH_4_/g VS_added_, whereas the processed food wastes, 100% PF and 100% GT, had a BMP of 466.2 ± 0.73 and 408.7 ± 6.6 mL_N_ CH_4_/g VS_added_, respectively, which is 1.42 and 1.12 times higher than 100% SS alone. For 100% SS, the biogas production started after 2 days and reached the first peak at day 8 with a rate of 21.5 mL_N_ biogas/g VS_added_·d (Figure 2(e)). The second peak occurred at day 17 with a peak value of 46.1 mL_N_ biogas/g VS_added_·d and after 21 days slowly decreased. Both food wastes started biogas production after day one and reached the first peak at day 17 with daily biogas yields of 54.3 and 45.4 mL_N_ biogas/g VS_added_·d, respectively, for PF and GT. The second peak values were 56.3 and 25.3 mL_N_ biogas/g VS_added_·d for PF and GT, respectively, at days 28 and 36. The technical digestion time, that is, T_80–90_ (the time for 80–90% of the maximum biogas production), was calculated to be between 20 and 27, 31 and 35, and 37 and 40 days for SS, PF, and GT, respectively. The technical digestion time can be used as a HRT for continuous anaerobic digestion for these substrates [24].

The codigestion of SS with PF enhanced the BMP from 199.6 ± 20.6 to 616.8 ± 30.2 mL_N_ CH_4_/g VS_added_ for PF fractions of 1% to 50%, that is, 4% to 287% increase in methane yield compared with 100% SS alone (Figure 1(b)). However, with 1% PF to 10% PF incorporation, a lag phase of 2 days was observed, and the 25–50% PF mixture with SS immediately started biogas production. For 1% PF to 25% PF, a single peak in daily biogas yield was observed at day 17 with peak values of 36.4 ± 0, 43.9 ± 7.1, 67.2 ± 4, and 86.7 ± 2.7 mL_N_ biogas/g VS_added_·d, respectively, for 1% PF, 2% PF, 10% PF, and 25% PF (Figure 1(f)). A rising trend was observed in peak value with increasing PF ratio. The production of biogas was decreased after 20 days and almost ceased after 36 days. However, for 50% PF, an inhibition in biogas production was obtained with two peaks. At day 15, the first peak of 42.4 ± 0 mL_N_ biogas/g VS_added_·d with easily degradable organic materials was noted and, at day 28, a small second peak of 29.6 ± 8.7 mL_N_ biogas/g VS_added_·d with slow degradation were observed. T_80–90_ was calculated between 20 and 26, 21 and 27, 21 and 27, 21 and 27, and 27 and 33 days, respectively, for 1%–50% PF incorporation.

SS mixed with GT enhanced the BMP of SS from 200 ± 2.6 to 561.3 ± 16.9 mL_N_ CH_4_/g VS_added_, that is, there was a 5% to 260% increase in methane production, by adding up to 50% GT during codigestion (Figure 1(c)). It was observed that increasing the GT fraction in the feedstock from 1% to 50% caused an increase in BMP up to 17 days and it started decreasing until completely ceased at around 36 days (Figure 1(g)). The peak values were 47.3 ± 2.4, 47.7 ± 3.1, 42.3 ± 1.8, 75.4 ± 13.8, and 89.8 ± 22.8 mL_N_ biogas/g VS_added_·d, respectively, for 1% GT, 2% GT, 10% GT, 25% GT, and 50% GT. No inhibition was observed with T_80–90_ between 20 and 26, 20.5 and 26, 21 and 28, 21 and 28, and 25 and 32 days, respectively, for 1%–50% GT incorporation.

For three substrates, biogas production improved with up to 50% FW addition (632.8 ± 10.1 mL_N_ CH_4_/g VS_added_) and decreased for the mixture ratio of 66.7% FW (603.3 ± 6.7 mL_N_ CH_4_/g VS_added_) (Figure 1(d)). An early peak at day 8 was observed for 5% FW with a peak value of 40.7 ± 7.9 mL_N_ biogas/g VS_added_·d. It was, however, observed at day 17 for 20–66.7% FW with peak values of 63.6 ± 0.5, 74.8 ± 6.8 and 73.3 ± 0 mL_N_ biogas/g VS_added_·d, respectively (Figure 1(h)). T_80–90_ was calculated between 24 and 35, 25 and 32, 24 and 30, and 26 and 37 days, respectively, for 5%–66.7% FW incorporation.

Therefore, the addition of FW with SS decreased the technical digestion time with a single peak. The VAs usually associated with the GT appear to be below inhibition up to 50%. However, the inhibition effect at 50% PF indicates that there is NH_4_ that reached a threshold (2.1 ± 0.1 g/L). Ammonia which is an important indicator of AD, is produced by the hydrolysis of proteins and urea [25, 26] and accumulates in the AD process [27]. FW was incorporated with SS.

The BMP assay can be utilised to calculate the synergic effect of codigestion as additional methane yield over the weighted average of the individual feedstock's methane yield [28]. The weighted experimental methane was calculated from single substrate using the following formulas

(1) Weighted  EMYFW=EMY100% SS∗P100% SS+EMY100% PF∗P100% PF+EMY100% GT∗P100% GT,

(2) Weighted  EMYPF=EMY100% SS∗P100% SS+EMY100% PF∗P100% PF,

(3) Weighted  EMYGT=EMY100% SS∗P100% SS+EMY100% GT∗P100% GT,

where weighted EMY_FW_, EMY_PF_, and EMY_GT_ represent the weighted average of the experimental methane yield of the substrates FW, PF, and GT, respectively. *P*_100% SS_, *P*_100% PF_, and *P*_100% GT_ refer to the percentage composition and EMY_100% SS_, EMY_100% PF_, and EMY_100% GT_ are the experimental methane yield for substrates SS, PF, and GT, respectively, in the cosubstrates mixture. According to Li et al. (2013) if the difference (EMY − weighted EMY) was higher than the standard deviation of EMY, synergic effect could be observed [29]. The EMYs of the codigestion substrates during the digestion period were analysed statistically with respect to the EMYs of 100% SS. As Table 3 shows, 1-2% PF and GT did not have very significant synergistic effects; however, increasing the amount of food wastes resulted in a very significant (*p* < 0.05) increase in methane yield compared to the digestion of SS alone.

A synergic effect was found in almost all of the cases when food wastes were added to SS representing higher biodegradability. This is possibly due to the adjustment in C/N ratios during codigestion [29] compared to the single substrate. The C/N ratio is a good indicator of the efficiency of AD that can be limited by inadequate amount and diversity of waste from a single resource. For example, high carbon content of a sample can cause rapid acidification and methanogenesis will be inhibited by the low pH. The optimum C/N ratio is waste specific over a range from 9 to 30 [31]. The C/N ratio of SS used in this study was 8.16 which is lower than the C/N ratio of PF and GT (17.64 and 15.5, resp.). Incorporating 50% FW in the feedstock with SS increased the C/N ratio of the reactors up to 12-13. Antagonism (probably due to inhibition) was observed for 50% PF. In case of three substrates, 5% FW showed the highest increase in methane yield. Luostarinen et al. (2009) also reported inhibition with the addition of grease trap sludge to SS of more than 50% [17]. However, these inhibitory effects were only deduced from the pattern of methane production and the synergistic effects and will require further investigations.

**Figure 3:**
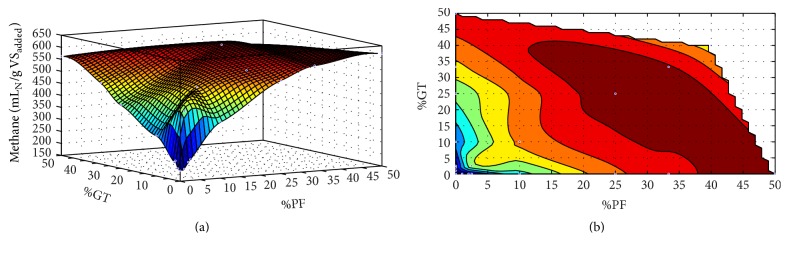
3D prediction of optimum FW incorporation: (a) surface plot and (b) contour plot.

To investigate the optimum mixture ratio of FW and SS with respect to methane yield, a trend was predicted using MATLAB (Figures 2(a)–2(c)). The *R* ^2^ correlation values were 0.999, 0.993, and 0.885 for %PF, %GT, and %FW incorporation with SS, respectively, indicating a good fit between experimental and predicted values. The results showed that methane yield obtained maximum values of 614.6, 562, and 651.1 mL_N_ CH_4_/g VS_added_ when 47% PF, 61.4% GT, and 48% FW were incorporated with SS improving the C/N ratio of 12.5.  Figure 3 shows the 3D model of optimum FW incorporation with SS, where %PF and %GT with SS on the *x*- and *y*-axis with methane yield on *z*-axis. The dark red area represents the maximum methane yield region. FW incorporation up to 48% with the mixture of GT and PF according to the dark red region will produce the maximum volume of biogas. Considering SS as the main substrate, batch experiments indicated that mixtures of more than 50% of SS with other substrates can be performed with no risk of inhibition. However, inhibition under continuous operation of a plant also depends on factors such as organic loading rate (OLR), HRT, and reactor configurations. Therefore, a small pilot scale continuousy fed anaerobic digestor should be operated before incorporating the mixture ratio.

### 3.2. Semicontinuous Experiments

**Figure 4:**
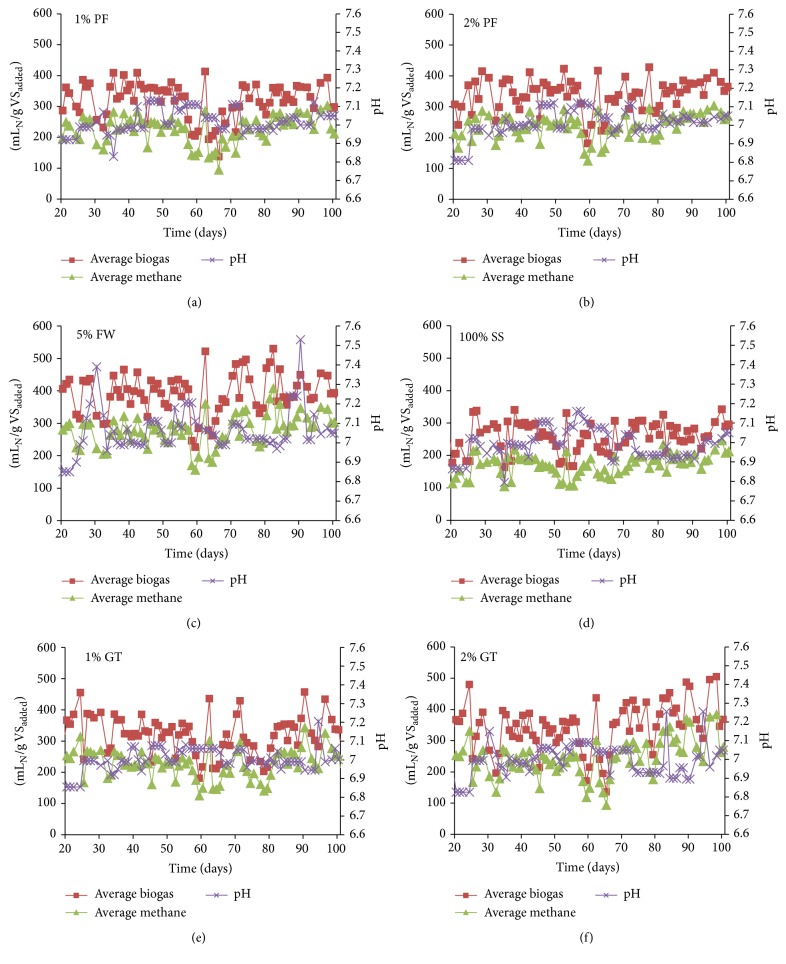
Daily biogas production, methane yield, and variation in pH during the codigestion of MWTP sludge with food wastes at different mix ratios: (a) 1% PF, (b) 2% PF, (c) 5% FW, (d) 100% MS, (e) 1% GT, and (f) 2% GT.

According to the requirement of the plant only 5% or less food waste incorporation was tested for process performances under semicontinuous conditions for six HRT cycles of 20 days each.  Figure 4 shows the specific biogas and methane production from the four cycles (20–100 days) reported as mL_N_/g VS_added_ fed to the reactor. The average daily methane yield from SS (100% SS) and different mixture ratios of SS with PF and GT (1% to 2%) varied between 212 and 415 mL_N_/g VS_added_. For small amounts of FW incorporation, biogas production was proportional to the percentage of FW and the biogas yield for 5% FW was the highest throughout the experiment duration which is consistent with the BMP assays [11].

For 100% SS, the average SBP was 284 ± 9.7 mL_N_/g VS with methane content in the range of 64% and 66%. The average TS, VS, and tCOD removal for 100% SS was 41%, 50%, and 58%, respectively, which was in agreement with COD and VS removal of 35% and 36%, respectively, reported by Silvestre et al. for continuous AD of sludge mix of 70% PS and 30% WAS at an OLR of 1.5 to 1.7 kg VS/m^3^·d and HRT of 20 days [9]. A low SBP of 236 ± 6.6 mL_N_/g VS was observed during the third HRT cycle (40–60 days) compared to HRT cycle two (20–40 days) when a new batch of feed was prepared with newly collected sample. Low TS, VS, and tCOD removal was also found during the period. This lag phase might be because of the biomass adaptation with the new feed [9]. The pH varied between 6.9 and 7.1 during the whole experiment.

The average SBP of 1% PF and 2% PF was 359 ± 9 and 367 ± 11 mL_N_/g VS_added_ which is 25% and 32% higher than the SBP from 100% SS alone. Similarly, 23% and 47% increase in SBP were observed for 1% GT and 2% GT with an average SBP of 355 ± 9 and 367 ± 3 mL_N_/g VS_added_. As FOG has high biodegradability and BMP value (when added below 20% of the influent COD) [13], codigestion with a small proportion of GT produced more biogas than the other food wastes in the same amount. The codigestion of three wastes SS : PF : GT at 95 : 2.5 : 2.5 (5% FW) produced an average SBP of 424 ± 10 mL_N_/g VS (methane yield 327 mL_N_/g VS) which is 50% higher than 100% SS (single substrate). These results are in agreement with the results reported by Luostarinen et al. (2009) [17] and Davidsson et al. (2008) [32]. They worked with SS and grease trap sludge (95 : 5 w/w) and reported methane yield of 374 and 295–308 mL/g VS corresponding to the organic loading of 1.67–2.23 and 2.5 kg VS/m^3^·d for HRT of 16 and 13 days, respectively. The addition of food wastes also increased the methane content and the average methane content was 69–72% in this experiment.

The TS removal for 1-2% food wastes (GT, PF) was between 42% and 49% and the corresponding VS removal was found to be between 50% and 56% (Table 4). This is similar to the VS removal reported in previous studies [17, 32]. At the start of the second HRT cycle (20–40 days), the pH was between 6.8 and 6.9 for all the reactors, possibly because of high VA production at the beginning. The pH started increasing after that, indicating the consumption of produced VA due to acidification and inoculum acclimatisation [33]. However, when a new feed was prepared in the fourth HRT cycle (60–80 days), a lag phase was observed with low organic content removal, and low pH as well as low biogas production. However after the lag phase the reactors produced stable biogas production in the last two HRT cycles of the codigestion.

Methane production was increased significantly from 2% GT after the lag phase, possibly because the methanogens were acclimated to inoculum [4]. However, GT which is mainly lipid-rich material [34] has been found to have wide variation in characteristics (from Table 1, where characteristics' results from two different sample collections are shown).

The daily biogas production was observed to fluctuate, although the feedstocks were prepared by homogenising to constant TS loading throughout the experiments. As the FW had high variations in their characteristics, feeding a very small proportion in the reactors every day (from a batch of prepared feedstock) resulted in variations. Therefore, the average biogas production over each HRT cycle is shown in Table 4 and a rising trend was observed because of the acclimatisation of the inoculum to the feedstock.

The biogas and methane production potential of the food wastes was very high because of its high fat and protein content. Therefore, the incorporation of FW at very small ratios (1–5%) with SS in codigestion significantly improved biogas production from the SS alone. Although the biogas production improved greatly, VS and COD removal was not improved significantly (Table 4). This was likely due to the huge amount of more slowly degradable and/or inert material in the SS (60% degradable) [17]. The biodegradability of FW on the other hand was probably close to 100% due to the dilution with SS which caused the high biogas production.

Although SBP and methane yield depend on the origin of the substrates, composition, and operational conditions (SRT, temperature), the results reported by Silvestre et al. (2011) [9] and Davidsson et al. (2008) [32] showed a methane yield lower than this study when a small percentage of wastes from the dissolved air flotation unit of a wastewater treatment plant and kitchen grease wastes were added to SS. In addition to the biogas yield, different parameters were monitored at the end of each cycle to assess the quality of the supernatant and digestate (Table 5). It was observed that the pH value remained relatively stable at around 7 throughout the operation of the reactors. The alkalinity in all the reactors was around 2.5 to 2.75 g/L which also indicates no accumulation of VAs and the highest VA was observed from 2% GT (0.315 g/L) which is well below the threshold of inhibition (4 g/L) [35]. The VA accumulation might cause the instability of the process and an inhibition of acetotrophic methanogenesis [13]. However, the VAs in the reactors indicate stable process conditions. Luostarinen et al. (2009) observed total VA accumulation of not more than 0.43 g/L with a high ratio of grease trap to sludge in the feedstock (71% of the feed VS) while working with a mixture of PS, WAS, and grease trap sludge [17]. The ammonia-N content in all the reactors was between 0.6 and 0.71 g/L which is below the inhibition range (1.5–2.0 g/L) [4].

The last aspect to consider in anaerobic codigestion is the possibility of producing high quality compost (or fertilizer). In this case, the dewatered digestate characteristics for heavy metal contents need to be considered when assessing the effect of codigestion [7].

In Australia, the concentration of contaminants present in the biosolids and the microbial quality are two important parameters for biosolids' classifications. Contaminant grade (C1 and C2) and treatment grade (T1, T2, and T3) are the classifications of biosolids based on the factors described where C1/T1 are high quality products and can be used without restriction. According to the EPA guideline, biosolids from wastewater treatment plants are categorised as C2/T3 [30]. Integration among the AD and composting is possible where composting can play the role of curing step to overcome the phytotoxicity limit for VA and ammonia [36]. In the AD reactors, no inhibition of VA and ammonia N was observed. The digestate characteristics were adequate for the production of good quality compost by integrating a simple aerobic poststabilisation and dewatering for biological stability.

In Australia, the regulation of heavy metals in fertilizers of organic origin is governed by the Fertilizer Working Group, Department of Agriculture, AU Government (http://www.agriculture.gov.au/). The concerned heavy metals are zinc (Zn), copper (Cu), nickel (Ni), cadmium (Cd), lead (Pb), chromium (Cr), and mercury (Hg) and their allowable limits are shown in Table 6.

These heavy metals may be present in concentrations above the legal limits which can potentially harm environment and affect crop quality, crop yield, and soil fertility. Heavy metal concentration may increase during AD due to the microbial mineralization and loss of volatile solids [37]. Most national regulations prohibit the use of organic fertilizers, for example, digestate, if the concentrations of one or more heavy metals are higher than the threshold concentrations. There is also evidence suggesting that AD increases the complexation of heavy metals with organic ligands and hence lowers the mobility of heavy metals in the digestate [38, 39]. However, the metal contents found in these experiments were less than the allowable limit used in Australia for high quality amendments. Table 6 shows the concentration of heavy metal in the digestate collected reactors after six HRT cycles (at the end of the experiment).

## 4. Conclusions

FW is a suitable cosubstrate for the anaerobic codigestion of SS. The addition of 5% FW to the SS increased the SBP by up to 50% during semicontinuous experiments. Although the TS, VS, and tCOD removal slightly increased with codigestion, the methane content of the bioga improved significantly. The reactors showed stable pH and performance with no inhibitory effect. Based on the results from batch assays and the use of surface modelling, FW can be added at ratios up to 47%-48% (v/v) without inhibition to the AD process. Overall these results reveal the high potential of codigestion FW with SS to enhance bioga yield and quality.

## Figures and Tables

**Table 1:** Characteristics of substrates and inoculum.

Parameters	Unit	SS	PF	GT		Inoculum
				1st sample	2nd sample	
TS	%	3.7 ± 0.1	18.77 ± 0.8	7 ± 0.2	26.1 ± 0.2	1.85 ± 0.2
VS	%	3.13 ± 0.11	18.06 ± 0.7	6.8 ± 0.16	25.55 ± 0.2	1.32 ± 0.12
tCOD	g/L	53.73 ± 8.2	239.1 ± 0.91	405.3 ± 50	475.5 ± 10	12.9 ± 2.8
sCOD	g/L	3.95 ± 0.6	3.42 ± 0.04	2.98 ± 0.9	3.8 ± 0.7	1.4 ± 0.7
Total N	g/L	2.6 ± 0.1	3.55 ± 0.15	3.5 ± 0.2	3.54 ± 0.2	1.86 ± 0.003
Ammonium	g/L	0.11 ± 0.01	0.11 ± 0.003	0.14 ± 0.01	0.26 ± 0.007	0.48 ± 0.007
Total PO_4_^3-^	g/L	1.5 ± 0.05	1.1 ± 0.04	2.56 ± 0.06	2.58 ± 0.1	0.9 ± 0.3
Total VA	g acetic acid/L	0.6 ± 0.01	1.98 ± 0.15	1.9 ± 0.2	2.03 ± 0.2	0.17 ± 0.013
Alkalinity	g CaCO_3_/L	2.7 ± 0.001	1.42 ± 0.001	1.3 ± 0.001	2.1 ± 0.01	4.1 ± 0.002
pH		6.36 ± 0.09	5.54 ± 0.01	5.0 ± 0.6	6 ± 0.3	7.55 ± 0.13

**Table 2:** Composition of the feedstocks used in the BMP and semicontinuous tests.

Experiment type	Substrates in feedstock	Substrates	Composition (w/w)	Nomenclature
Batch	Single	SS	100	100% SS
		PF	100	100% PF
		GT	100	100% GT

Batch	Two	SS : PF	99 : 01	1% PF
		SS : PF	98 : 02	2% PF
		SS : PF	90 : 10	10% PF
		SS : PF	75 : 25	25% PF
		SS : PF	50 : 50	50% PF
		SS : GT	99 : 01	1% GT
		SS : GT	98 : 02	2% GT
		SS : GT	90 : 10	10% GT
		SS : GT	75 : 25	25% GT
		SS : GT	50 : 50	50% GT

Batch	Three	SS : PF : GT	95 : 2.5 : 2.5	5% FW^#^
		SS : PF : GT	80 : 10 : 10	20% FW^#^
		SS : PF : GT	50 : 25 : 25	50% FW^#^
		SS : PF : GT	33.3 : 33.3 : 33.3	66.67% FW^#^

Semicontinuous	Single	SS	100	100% SS

Semicontinuous	Two	SS : PF	99 : 1	1% PF
		SS : PF	98 : 2	2% PF
		SS : GT	99 : 1	1% GT
		SS : GT	98 : 2	2% GT

Semicontinuous	Three	SS : PF : GT	95 : 2.5 : 2.5	5% FW^#^

^#^FW = mixture of PF and GT at ratio 50 : 50 (w/w).

**Table 3:** Synergistic effect evaluation of codigestion of SS with PF, GT, and FW (mixture of PF : GT).

Substrates ratio^a^	EMY	SD	Weighted EMY	Difference	Increase in EMY (%)	*p* value	Synergistic effect
1% PF	199.6	20.6	195.7	3.9	2.0	0.9310	Not clear
2% PF	226.6	16.3	198.4	28.2	14.2	0.6106	Not significant
10% PF	383.1	22.9	220.3	162.8	73.9	0.0462	Synergistic
25% PF	537.5	12.3	261.3	276.2	105.7	0.0084	Synergistic
50% PF	616.8	30.2	329.6	287.2	87.2	0.0066	Synergistic
1% GT	200.8	2.6	195.1	5.16	2.7	0.9067	Not clear
2% GT	230.6	10.3	197.3	33.32	16.9	0.5423	Not significant
10% GT	317.3	14.8	214.5	102.8	47.9	0.0467	Synergistic
25% GT	413.2	10.1	246.7	166.3	67.4	0.0259	Synergistic
50% GT	561.3	16.9	300.8	260.5	86.6	0.0081	Synergistic
5% FW	433.7	72.7	205.2	228.5	111.4	0.0176	Synergistic
20% FW	508.9	70.1	241.8	267.1	110.4	0.0110	Synergistic
50% FW	632.8	16.1	315.2	317.6	100.8	0.0038	Synergistic
66.67% FW	603.3	6.7	352.4	250.9	71.2	0.0066	Synergistic

EMY: experimental methane yield (mL/g VS_added_); SD: standard deviation; and weighted EMY: weighted average of experimental methane yield for cosubstrates.

^a^Percentage of food wastes (PF, GT, and FW) mixed with SS.

**Table 4:** Biogas production and process performance in terms of TS, VS, and COD removal.

Feedstocks	Parameters	Period I (0–20 d)	Period II (20–40 d)	Period III (40–60 d)	Period IV (60–80 d)	Period V (80–100 d)	Period VI (100–120 d)
1% PF	Avg biogas	256 ± 16	337 ± 14	320 ± 1.5	284 ± 8	339 ± 7	355 ± 9
	CH4%		69 ± 3.2	65 ± 7.81	69 ± 2.8	77 ± 2.8	71 ± 6.7
	TS removal%	43 ± 0.01	43 ± 0.03	40 ± 0.04	40 ± 0.4	46 ± 1	41 ± 4.5
	VS removal%	49 ± 0	51 ± 0.02	49 ± 0.03	45 ± 0.1	57 ± 3.3	50 ± 2.1
	COD removal%	61 ± 0.06	58 ± 0.01	53 ± 0.02	59 ± 0.03	59 ± 0.03	55 ± 2.03
	pH	7.4 ± 0.4	7.0 ± 0.08	7.1 ± 0.08	7.01 ± 0.06	7.02 ± 0.05	7.07 ± 0.03

2% PF	Avg biogas	252 ± 14	335 ± 9	334 ± 2	322 ± 1	364 ± 2	367 ± 3
	CH4%		69 ± 2.4	66 ± 3.4	69 ± 2.4	74 ± 1.4	69 ± 5.4
	TS removal%	43 ± 0.02	45 ± 0.01	41 ± 0.05	45 ± 2.5	46 ± 0.06	45 ± 2.2
	VS removal%	51 ± 0.01	52 ± 0.01	50 ± 0.04	52 ± 0.05	55 ± 0.03	53 ± 1.6
	COD removal%	58 ± 0.1	54 ± 0.01	55 ± 0.06	59 ± 0.03	57 ± 0.03	57 ± 2.05
	pH	7.3 ± 0.4	7 ± 0.04	7.1 ± 0.06	7.01 ± 0.01	7.03 ± 0.02	7.05 ± 0.04

5% FW	Avg biogas	281 ± 1	376 ± 2	386 ± 3	393 ± 2	415 ± 19	424 ± 10
	CH4%		69 ± 5.5	68 ± 7.5	69 ± 7.8	77 ± 4.9	72 ± 5.1
	TS removal%	49 ± 0.01	52 ± 0.02	52 ± 0.08	44 ± 0.06	50 ± 1.0	52 ± 0.07
	VS removal%	56 ± 0.01	60 ± 0.02	60 ± 0.02	55 ± 0.08	54 ± 1.01	59 ± 4.05
	COD removal%	58 ± 0.1	54 ± 0.01	55 ± 0.02	60 ± 0.04	54 ± 0.08	58 ± 1.01
	pH	7.3 ± 0.5	7.09 ± 0.06	7.08 ± 0.08	7.05 ± 0.07	7.1 ± 0.3	7.05 ± 0.04

1% GT	Avg biogas	253 ± 36	285 ± 15	308 ± 7	348 ± 5	345 ± 12	361 ± 1
	CH4%		69 ± 2.51	67 ± 2.3	69 ± 2.12	75 ± 2.8	69 ± 7.1
	TS removal%	44 ± 0.01	45 ± 0.01	45 ± 0.06	43 ± 0.01	44 ± 0.06	45 ± 3.05
	VS removal%	52 ± 0.01	52 ± 0.02	46 ± 0.06	50 ± 1.02	48 ± 0.04	48 ± 5.09
	COD removal%	63 ± 0.04	59 ± 0.01	63 ± 0.04	65 ± 1.06	56 ± 2.3	59 ± 1.06
	pH	7.3 ± 0.5	7 ± 0.06	7.02 ± 0.04	7 ± 0.05	7 ± 0.12	7.08 ± 0.13

2% GT	Avg biogas	284 ± 9	336 ± 5	312 ± 3	329 ± 7	405 ± 4	395 ± 8
	CH4%		68 ± 2	66 ± 5.1	69 ± 4.2	76 ± 4.9	72 ± 4.0
	TS removal%	46 ± 0.02	45 ± 0.01	46 ± 0.04	45 ± 0.01	44 ± 0.02	46 ± 2.04
	VS removal%	54 ± 0.01	54 ± 0.02	53 ± 0.03	53 ± 0.01	57 ± 0.08	53 ± 0.03
	COD removal%	65 ± 0.06	60 ± 0.01	60 ± 0.05	60 ± 0.04	56 ± 0.01	59 ± 0.04
	pH	7.25 ± 0.42	7 ± 0.09	7.03 ± 0.05	7 ± 0.06	7.01 ± 0.2	7.06 ± 0.07

100% SS	Avg biogas	212 ± 1.7	271 ± 5.8	236 ± 6.6	264 ± 3.16	269 ± 3.5	284 ± 9.7
	CH4%		64 ± 4.5	62 ± 1.5	64 ± 7.8	66 ± 2.8	66 ± 9.6
	TS removal%	43 ± 0.02	41 ± 0.03	40 ± 0.06	40 ± 0.01	39 ± 0.03	40 ± 6.08
	VS removal%	50 ± 0.01	54 ± 0.02	46 ± 0.06	50 ± 1.3	51 ± 2.6	53 ± 1.7
	COD removal%	60 ± 0.03	58 ± 0.03	55 ± 0.06	56 ± 0.6	55 ± 0.8	55 ± 1.01
	pH	7 ± 0.12	6.99 ± 0.09	7.04 ± 0.09	6.93 ± 0.02	7.03 ± 0.05	7.05 ± 0.04

**Table 5:** Bench scale AD reactors' performance at the end of the experiment.

Parameter	Unit	1% PF	2% PF	5% FW	1% GT	2% GT	100% SS
TS	g/L	21.15 ± 2.43	20.62 ± 2.57	21.13 ± 5.1	20.02 ± 3.46	20.45 ± 0.26	20.34 ± 0.97
VS	g/L	15.67 ± 2.1	17.72 ± 1.61	17.16 ± 1.97	15.33 ± 0.28	14.61 ± 0.26	14.90 ± 2.92
tCOD	g/L	28.025 ± 0.25	26.65 ± 0.07	28.9 ± 0.21	26.075 ± 7.88	28.55 ± 0.21	29.025 ± 5.69
sCOD	g/L	2.05 ± 0.10	1.765 ± 0.06	3.24 ± 0.10	1.755 ± 0.02	2.285 ± 0.04	1.925 ± 0.11
TS removal	%	45 ± 2	47 ± 3	52 ± 5	48 ± 3	48 ± 1	46 ± 1
VS removal	%	52 ± 2.1	53 ± 1.6	55 ± 2	53 ± 0.3	57 ± 0.3	53 ± 3
COD removal	%	57 ± 2.5	59 ± 0.7	59 ± 2.1	60 ± 7.8	59 ± 2.1	55 ± 5.7
TP	g/L	0.36 ± 0.03	0.38 ± 0.04	0.44 ± 0.04	0.4 ± 0.02	0.42 ± 0.01	0.41 ± 0.001
TN	g/L	0.93 ± 0.035	0.895 ± 0.04	0.98 ± 0.035	0.877 ± 0.018	0.965 ± 0.014	0.945 ± 0.035
TKN^*∗*^	g/L	1.9 ± 0.3	2 ± 0.42	2.4 ± 0.3	2.2 ± 0.2	2.3 ± 0.15	2.2 ± 0.3
NH4-N	g/L	0.62 ± 0.014	0.575 ± 0.035	0.685 ± 0.05	0.7 ± 0.014	0.71 ± 0.014	0.67 ± 0.014
VA	g/L	0.147 ± 0.004	0.148 ± 0.01	0.266 ± 0.07	0.253 ± 0.05	0.315 ± 0.014	0.208 ± 0.005
pH		7 ± 0	7.08 ± 0	7.13 ± 0.02	7.05 ± 0.02	7.09 ± 0.05	7.04 ± 0.02
Alkalinity	g/L	2.498 ± 0.29	2.7 ± 0.06	2.756 ± 0.08	2.711 ± 0.05	2.678 ± 0.03	2.671 ± 0.05

^*∗*^Analyses were carried out at a commercial laboratory (ALS, Australia).

**Table 6:** Digestate heavy metals concentration^**∗**^ in mL/g at the end of the experiment (after six HRT cycle of 20 days).

Parameter	1% PF	2% PF	5% FW	1% GT	2% GT	100% SS	Limit^**∗∗**^ (mg/kg)
Ca	430 ± 4	455 ± 5	480 ± 6	450 ± 5	440 ± 4	450 ± 7	
Mg	82 ± 1	85 ± 0.5	89 ± 0.7	89 ± 0.4	85 ± 0.3	87 ± 0.5	
Ca hardness	1100 ± 2	1100 ± 0.5	1200 ± 1	1100 ± 0	1100 ± 0.5	1100 ± 0	
Mg hardness	340 ± 0	350 ± 0	370 ± 1	360 ± 2	350 ± 5	360 ± 5	
Al	170 ± 0	170 ± 0	180 ± 0	170 ± 0	170 ± 0	200 ± 0	
As	<1	<1	<1	<1	<1	<1	20
Cd	<0.1	<0.1	<0.1	<0.1	<0.1	<0.1	1
Cr	0.5 ± 0.1	0.5 ± 0.1	0.5 ± 0.1	0.5 ± 0.1	0.5 ± 0.1	0.5 ± 0.1	400
Cu	11 ± 1	11 ± 1	12 ± 0	12 ± 1	12 ± 0	14 ± 0	100
Fe	160 ± 10	160 ± 5	170 ± 10	170 ± 10	170 ± 5	170 ± 5	
Pb	<0.5	<0.5	<0.5	<0.5	0.5 ± 0.1	0.6 ± 0.1	300
Hg	<1	<1	<1	<1	<1	<1	1
Ni	0.4 ± 0.1	0.4 ± 0.1	0.5 ± 0.1	0.4 ± 0.1	0.4 ± 0.1	0.4 ± 0.1	60
Zn	19 ± 0.5	20 ± 0.5	21 ± 1	20 ± 0.5	21 ± 1	22 ± 1.5	200
Si	170 ± 5	180 ± 5	170 ± 5	170 ± 5	180 ± 5	210 ± 10	
Si-SiO2	350 ± 10	380 ± 5	370 ± 5	370 ± 5	380 ± 5	440 ± 10	
S	160 ± 5	170 ± 0	180 ± 5	170 ± 5	170 ± 5	200 ± 5	
S-SO4	480 ± 10	520 ± 5	540 ± 5	520 ± 5	520 ± 5	600 ± 10	

^**∗**^Heavy metal screening of the digestate samples was carried out by a commercial laboratory (ALS Environmental Division: Water Research Group).

^**∗∗**^Contaminant upper limits for biosolids as grade C1 [30].
